# Time indices of pre-hospital EMS missions before and during the COVID-19 pandemic: a cross-sectional study in Iran

**DOI:** 10.1186/s12873-023-00780-3

**Published:** 2023-01-28

**Authors:** Mohammadreza Sabbaghi, Mohammad Namazinia, Kheizaran Miri

**Affiliations:** 1 Department of Medical Emergency, School of Nursing and Midwifery, Torbat Heydariyeh University of Medical Sciences, Torbat Heydariyeh, Iran; 2 Department of Nursing, School of Nursing and Midwifery, Torbat Heydariyeh University of Medical Sciences, Torbat Heydariyeh, Iran

**Keywords:** COVID-19, EMS, Time Indices

## Abstract

**Background:**

The COVID-19 pandemic resulted in many changes in pre-hospital emergency medical services (EMS), including wearing full-body protective suits and well-fitted face masks, which can influence time indices in the course of service delivery. The present study aimed to compare the time indices of pre-hospital EMS missions before and during the COVID-19 pandemic in Iran.

**Methods:**

This descriptive cross-sectional study used census sampling to select 17,860 emergency calls that caused patient transfer to medical facilities from March 2018 to March 2021 and then examined the time indices of pre-hospital EMS missions during the COVID-19 pandemic. The data collection tool was a two-part checklist: patients' individual characteristics and pre-hospital EMS mission time indices. The data were further analyzed using the SPSS16 and independent samples t-test.

**Results:**

Out of the patients transferred by the EMS, 11,773 cases (65.9%) were male and 6,087 (34.1%) were female. The most common reason for the emergency calls was accidents (28.0%). Moreover, response time (*P* < 0.001), on-scene time (*P* < 0.001), transfer time (*P* < 0.001), total run time (*P* < 0.001), and round trip time (*P* < 0.001) increased significantly during the COVID-19 pandemic.

**Conclusions:**

We concluded that the EMS time indices elevated following the COVID-19 pandemic. Updating pre-hospital information management systems, ambulances and medical equipment, as well as holding training courses for pre-hospital EMS personnel could effectively enhance the time indices of pre-hospital EMS missions.

## Background

Pre-hospital emergency medical services (EMS) are the main pillar of healthcare systems across the world [1] because they help patients transfer to medical facilities, deliver the right treatment at the right time in the right place, and exploit the available resources [2]. Pre-hospital EMS starts from the bedside and terminates in the emergency department [3].

A variety of indices evaluate pre-hospital EMS [4], including response time, lag time, on-scene time, transfer time, total run time, and round trip time. The lag time is the time interval between receiving an emergency call and dispatching an ambulance, We concluded that the EMS time indices elevated following the COVID-19 pandemic. Updating pre-hospital information management systems, ambulances and medical equipment, as well as holding training courses for pre-hospital EMS personnel could effectively enhance the time indices of pre-hospital EMS missions.

The response time is the time interval between the receipt of an emergency call and the ambulance arrival at the scene, and the scene time represents the time interval when the ambulance arrives and leaves the scene. The transfer time means the time interval between leaving the scene and arriving at the medical facilities, the total run time is the sum of response time, on-scene time, and transfer time, while the round trip time is the time interval between an ambulance dispatch from the base and its return to the base [5].

Some factors can affect the time indices of pre-hospital EMS missions [6], including the COVID-19 pandemic [7, 8]. Coronavirus has emerged as a global health threat due to its spread over the last two decades [9]. In accordance with the epidemiological data worldwide, the United States, India, and Brazil have been severely affected by the COVID-19, with over 663,020,790 people being infected on October 28, 2022, of whom more than 6,689,523 cases died. There were 7,560,947 confirmed cases in Iran, with 144,677 cases being dead due to the COVID-19 disease https://www.worldometers.info/coronavirus/.

The COVID-19 pandemic has thus posed unprecedented challenges to health care systems [10, 11], leading to compulsory changes in pre-hospital EMS protocols, such as wearing full-body protective suits and sell-fitted face masks, and pre-hospital disinfection protocols after contacts with patients [8, 12].

Murphy reported an increased time interval between the ambulance arrival at the scene and its exit from the scene during the COVID-19 pandemic [13]. Laukkanen also mentioned an increased total time of pre-hospital EMS missions during the pandemic [14].

About 25,000 EMS personnel provide services in Iran's Emergency Organization and there is one ambulance for every 50,000 people and 3,000 land bases, including 1,700 road bases and 1,300 urban bases, and 50 air bases.

The performance of pre-hospital EMS can play a leading role in community health and we found no attempt on pre-hospital EMS time indices during the COVID-19 pandemic in Iran, so this study aimed to compare the time indices of pre-hospital EMS missions before and during the COVID-19 pandemic in Iran.

## Methods

### Study setting and design

This descriptive cross-sectional study was conducted in Torbat-e Heydarieh, Khorasan Razavi, Iran, with a population of about one million inhabitants.

### Populations, inclusion and exclusion criteria

The study population was 17,860 patients transferred to medical facilities by EMS and the affiliated bases in Torbat-e Heydarieh from March 2018 to March 2022, who were selected by census sampling.

We considered March 1, 2018 to February 20, 2020 as before the COVID-19 pandemic, and February 21, 2020 to March 1, 2022 as during the COVID-19 pandemic.

### Instruments of measurement

The data collection tool was a researcher-made checklist about the time indices of pre-hospital EMS missions. It consisted of two parts: patients' individual characteristics (name, gender, age, place of residence, and main complaint), and pre-hospital EMS mission time indices (times receiving the mission, moving from the base, arriving at the scene, moving from the scene, reaching medical facilities, delivering EMS, terminating the mission and returning to the base). As shown in Table 1, the required time indices were calculated using the mission time data.

**Table 1:** Definition of time indices of prehospital emergency services

Indices	Definitions
Delay time	The time interval between receiving an emergency call and sending an ambulance
Response time	The time interval between reception of an emergency call and the arrival of the ambulance at the scene
On-scene time	The time interval between the arrival of the ambulance at the scene and its exit from the scene
Transport time	The time interval between ambulance exit from the scene and its arrival to the emergency department
Total run time	The total of 3 time periods of response, presence at the scene, and transfer to the hospital
Round trip time	The time interval between an ambulance dispatch from the base and its return to the base

This tool was used in several studies in Iran to investigate time indices [5, 15, 16]. Quantitative content validity index (CVI = 0.9) was thus utilized to determine the validity of this instrument in the present study, so ten faculty members of nursing and EMS evaluated this tool and provided some suggestions for the final tool. The reliability of this study was further confirmed by completing the time index tool for about 30 samples, with the Cronbach's alpha coefficient of 0.81 being calculated based on the internal consistency.

### Sampling

After receiving the code of ethics, the first author coordinated with the head of ASAYAR (the software-based pre-hospital information management system) headquarters, referred to the Pre-Hospital EMS headquarters in Torbat-e Heydariyeh, Iran to obtain the raw data. ASAYAR is responsible for managing and controlling the process of service delivery in the Emergency Organization from the moment the client calls until the end of the mission. This software has been in the Pre-hospital EMS headquarters, Torbat Heydariyeh, Iran since March 2018. Considering that the samples were taken from the ASAYAR system, there were no missing items in the sampling.

### Statistical analysis

The data were statistically analyzed using SPSS16. The descriptive statistics (frequency, mean, and standard deviation) were further used to describe and categorize the data, and the independent samples t-test was used to compare the time indices before and during the COVID-19 pandemic. The normality of the quantitative variables was further assessed by the Kolmogorov–Smirnov test. The confidence interval of 95% and the significance level of 0.05 were considered in all tests.

## Results

According to the data recorded in ASAYAR affiliated to Torbat-e Heydariyeh University of Medical Sciences, Pre-Hospital EMS headquarters received 231,682 calls from March 2018 to March 2021, of which 40,056 calls (17.2%) led to an ambulance dispatch to the emergency site, and of these, 17,860 (7.7% of the total calls and 44.5% of the calls resulting in the ambulance dispatch) missions caused patient transfer to the affiliated medical facilities. Among the missions ended in the medical facilities, 5,613 cases (32.4%) were before the COVID-19 pandemic, while 12,247 missions (68.5%) were during the COVID-19 pandemic.

The demographic studies showed that most of the patients transferred to the medical facilities before and during the COVID-19 pandemic were over 60 years old (31.1%). The results also revealed that most of the patients were male before and during the COVID-19 pandemic (65.9%), with most pre-hospital EMS missions being about accidents (28%). Cardiac disease, weakness and lethargy were the main reasons for patient transfer to medical facilities following accidents before and during the COVID-19 pandemic (Table 2).

**Table 2:** Demographic characteristics of the patients by time periods studied

Variables	Group		Total (*n* = 17,860)
	Before the Covid Pandemic- 19 (*n* = 5613)	During the Covid Pandemic – 19 (*n* = 12,247)	
Age, n (%)			
< 15	468(8.3)	833(6.8)	1301(7.3)
15- 30	1354(24.1)	2784(22.7)	4138(23.2)
30- 45	1178(21.0)	2662(21.7)	3840(21.5)
45- 60	911(16.2)	2115(17.3)	3026(16.9)
$\ge$ 60	1702(30.3)	3853(31.5)	5555(31.1)
Gender, n (%)			
Male	3715(66.2)	8058(65.8)	11,773(65.9)
Female	1898(33.8)	4189(34.2)	6087(34.1)
Diagnosis, n (%)			
Impaired consciousness	104(1.9)	1107(9.0)	1211(6.8)
Blood pressure emergencies	267(4.8)	403(3.3)	670(3.8)
Weakness and lethargy	534(9.5)	1068(8.7)	1602(9.0)
Toxication	397(7.1)	290(2.4)	687(3.8)
Respiratory emergencies	80(1.4)	855(7.0)	922(5.2)
Accident	1640(29.2)	3369(27.5)	5009(28.0)
Cardiovascular	626(11.2)	1150(9.4)	1774(9.9)
Beatings and injuries	133(2.4)	344(2.8)	477(2.7)
Suspected Covid-19	-	773(6.3)	786(4.4)
Organ trauma	58(1.0)	439(3.6)	497(2.8)
Fall	303(5.4)	691(5.6)	994(5.6)
Women's emergencies	60(1.1)	165(1.6)	225(1.3)
Neurological emergencies	370(6.6)	694(5.7)	1064(6.0)
Psychological emergencies	110(2.0)	197(1.6)	307(1.7)
Internal emergencies	340(6.1)	610(5.0)	950(5.3)
Electric shock and burns	19(0.3)	65(0.5)	84(0.5)
Sting	244(4.3)	15(0.1)	259(1.4)
Other	328(5.8)	12(0.1)	342(1.9)
Time of mission, n (%)			
Morning	1900(33.8)	4121(33.6)	6021(33.7)
Evening	1721(30.7)	3840(31.4)	5561(31.1)
Night	1992(35.5)	4286(35.0)	6278(35.2)

The response time in 65.3% of the EMS missions was under eight minutes before and during the COVID-19 pandemic. The transfer time in 75.8% of the missions was less than 10 min, while the total transfer time in 42.4% of the missions was less than 40 min (Table 3).

**Table 3:** Frequency distribution of patients based on pre-hospital emergency time indices by study time periods

Time Indices	Group			Total (*n* = 17,860)
	Before the Covid Pandemic- 19 (*n* = 5613)	During the Covid Pandemic- 19 (*n* = 12,247)		
Delay time, n (%)				
< 1	1332(23.7)	3302(27.0)	4634(25.9)	
1–2	3166(55.7)	6406(52.3)	9532(53.4)	
2–6	1155(20.66)	2539(20.7)	3694(20.7)	
Response time, n (%)				
8 ≥	4048(72.5)	7596(62.0)	11,664(65.3)	
9–16	1224(21.8)	3731(30.5)	4955(27.7)	
> 16	321(5.7)	920(7.5)	1241(6.9)	
On-Scene time, n (%)				
10 ≥	2745(48.9)	4965(40.5)	7710(43.2)	
11–20	2386(42.5)	5709(46.6)	8095(45.3)	
> 20	482(8.6)	1573(12.8)	2055(11.5)	
Transport time, n (%)				
10 ≥	4523(80.6)	9016(73.6)	13,539(75.8)	
11–20	729(13.0)	2077(17.0)	2806(15.7)	
> 20	361(6.4)	1154(9.4)	1515(8.5)	
Total run time, n (%)				
25 ≥	2804(50.0)	4394(35.9)	7198(40.2)	
26–35	1737(30.9)	4374(35.7)	6111(34.2)	
36–45	550(9.8)	1841(15.0)	2391(13.4)	
> 45	522(9.3)	1638(13.4)	2160(12.1)	
Round trip time, n (%)				
40 ≥	2924(52.1)	4655(38.0)	7579(42.4)	
41–50	1142(20.3)	2959(24.2)	4101(23.0)	
51–60	537(9.6)	1590(13.0)	2127(11.9)	
> 60	1010(18.0)	3043(24.8)	4053(22.7)	

**Fig. 1:**
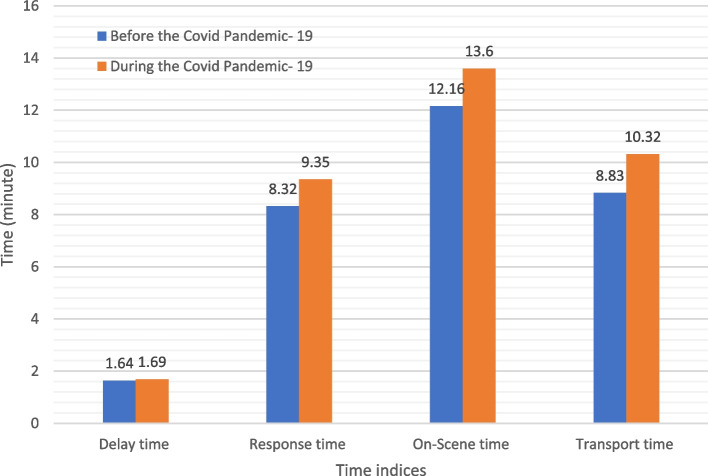
Prehospital emergency time indices by study intervals

Comparing the time indices before and during the COVID-19 pandemic showed that the response time (*p* < 0.001), on-scene time (*p* < 0.001), transfer time (*p* < 0.001), total run time (*p* < 0.001), and round trip time (*p* < 0.001) compounded significantly during COVID-19, but we found no difference in the lag time (*p* = 0.070) before and during the COVID-19 pandemic (Fig. 1) (Table 4).

**Table 4:** Prehospital emergency time indices by study intervals

Time Indices	Number	Mean ± SD	t	*P*
Delay time				
Before the Covid Pandemic- 19	5613	1.64 ± 1.5	-1.814	*P* = 0.070
During the Covid Pandemic- 19	12,247	1.69 ± 1.9		
Response time				
Before the Covid Pandemic- 19	5613	8.32 ± 8.5	-5.945	*P* < 0.001
During the Covid Pandemic- 19	12,247	9.35 ± 11.6		
On-Scene time				
Before the Covid Pandemic- 19	5613	12.16 ± 6.6	-11.852	*P* < 0.001
During the Covid Pandemic- 19	12,247	13.64 ± 7.2		
Transport time				
Before the Covid Pandemic- 19	5613	8.83 ± 8.6	-10.018	*P* < 0.001
During the Covid Pandemic- 19	12,247	10.32 ± 10.5		
Total run time				
Before the Covid Pandemic- 19	5613	29.32 ± 14.8	-14.830	*P* < 0.001
During the Covid Pandemic—19	12,247	33.15 ± 18.2		
Round trip time				
Before the Covid Pandemic- 19	5613	52.08 ± 47.2	-7.541	*P* < 0.001
During the Covid Pandemic- 19	12,247	57.83 ± 47.0		

Independent t-test

## Discussion

The present study aimed to compare the time indices of pre-hospital EMS missions before and during the COVID-19 pandemic. The results revealed that the response time, on-scene time, transfer time, total run time, and round trip time were significantly different.

The Delay time had no significant change before and during the COVID-19 pandemic. Eskol et al., Saffy et al., and Lim et al. supported our study and indicated no significant changes in the lag time before and during the COVID-19 pandemic [17–19]. Since noisy phone line and the Internet interference often affected the lag time [20], the COVID-19 pandemic had no effect on the lag time in the above-mentioned studies.

Our study showed a significant increase in the response time during the COVID-19 pandemic. Lim et al. (2020) agreed with us; they studied the effect of COVID-19 on out-of-hospital cardiac arrest in Singapore and indicated that the response time during COVID-19 had been longer than that before it [19]. Similar results might be because patient triage in the emergency department had been longer during the COVID-19 pandemic (taking a history of the COVID-19 symptoms and examining the patients in terms of travel to suspected areas). Eskol et al. (2020) compared the changes in the rate of EMS calls in southern Denmark before and during the COVID-19 pandemic but found no significant changes in the response time before and during the pandemic [17]. Moreover, Satty et al. (2021) investigated emergency responses to non-traffic accidents during the COVID-19 pandemic in Pennsylvania, the United States and found no changes in the response time before and during the pandemic [18], so Eskol et al. and Satty et al. did not support our results. One reason for this discrepancy was lack of personnel and equipment in Iran's Emergency Organization, leading to the poor performance of pre-hospital EMS bases. This could be due to noisy phone line, which in turn could result in late connection to ASAYAR.

We found a significant growth in the on-scene time during the COVID-19 pandemic. Murphy et al. examined the effect of COVID-19 on emergency responses in Japan and revealed that the on-scene time significantly increased during the COVID-19 pandemic [13]. Velasco et al. (2020) indicated an increase in that on-scene time for all patients during the pandemic [21]. Lim et al. examined the impact of COVID-19 on out-of-hospital cardiac arrest in Singapore and demonstrated that the on-scene time increased during the COVID-19 pandemic [19]. According to the guidelines declared by the World Health Organization in March 2020, the use of personal protective equipment (including face masks, gowns, gloves, and eye protectors) was an obligation while transferring the COVID-19 patients to medical facilities [22–24]. The pre-hospital EMS bases also provided such instructions, so the time spent using personal protective equipment was added to the on-scene time, but Javis et al. (2020) disagreed with us because they studied only on traffic-related trauma patients in the United States; various surveys indicated that urban traffic decreased during the COVID-19 pandemic [13, 25], thereby reducing the presence of pre-hospital EMS.

We reported a significant rise in the transfer time during the COVID-19 pandemic because the transfer of the COVID-19 patients took place in certain medical facilities, thus leading to a longer transfer time, which was inconsistent with the reports by Javis et al. (2020) [26] who conducted their study on traffic-related trauma patients, so transfer time was partly due to the social distancing protocols that reduced the number of people on the roads. Yu et al. (2021) evaluated EMS in stroke patients in Taiwan and observed no significant difference in the transfer time before and during the COVID-19 pandemic [27]. Ageta et al. (2020) studied the impact of the COVID-19 pandemic on emergency responses in Japan and found no significant difference in the transfer time before and during the disease [28].

The total run time in the present study increased significantly before and during the COVID-19 pandemic; Ageta et al. (2020) examined the impact of the COVID-19 pandemic on EMS in Japan and supported our results [28]. One of the reasons for such consistency was that the pre-hospital EMS personnel had to follow protective instructions against patients with COVID-19, such as wearing well-fitted face masks, gowns, gloves, etc., thereby increasing the total run time. Javis et al. found no significant difference in the total run time before and during the COVID-19 pandemic [26]. One reason for the discrepancy here was that Javis e t al. reflected on traffic-related trauma patients, so access to patients and their transfer to medical facilities were done in shorter times due to the low traffic volume during the COVID-19 pandemic.

We reported a significant increase in the round trip time before and during the COVID-19 pandemic; Laukkanen et al. (2021) and Prezant et al. (2020) examined the impact of COVID-19 on pre-hospital EMS missions and confirmed our results [14, 29]. The new health protocols, the use of personal protective equipment and disinfection of ambulances and equipment had further added to this time, so their results were in harmony with the findings in the present study.

This study had several limitations: first, it was limited to Torbat Heydarieh city in Iran. Second, Internet disruption could affect time indicators. Third, some special incidents caused many injuries and led to the dispatch of auxiliary ambulances, fire brigades, and police, which could have an effect on the time indicators.

## Conclusion

The study results showed an increase in the EMS time indices during the COVID-19 pandemic. Given many missions accomplished by the pre-hospital EMS personnel during the COVID-19 pandemic, the pre-hospital EMS officials should take effective steps to improve such time indices by updating pre-hospital information management systems, upgrading ambulances and medical equipment, and holding relevant training courses for the personnel to boost the quality of patient care during the COVID-19 pandemic and similar conditions.

## Data Availability

The datasets generated during the current study are available in the [spss] repository, [https://doi.org/10.6084/m9.figshare.20263680].

## Abbreviations

EMS: Emergency medical services
DASS-21: Depression, Anxiety, and Stress Scale
